# Medication adherence in type 2 diabetes mellitus patients during Covid-19 pandemic: a cross-sectional study from the United Arab Emirates

**DOI:** 10.12688/f1000research.51729.2

**Published:** 2021-06-25

**Authors:** Ameena Asheq, Akram Ashames, Moawia Al-Tabakha, Nageeb Hassan, Ammar Jairoun

**Affiliations:** 1 College of Pharmacy and Health Sciences, Ajman University, 346 Ajman, Ajman, United Arab Emirates; 2 Center of Medical and Bio-allied Health Sciences Research, Ajman University, 346 Ajman, Ajman, United Arab Emirates; 3 Health and Safety Department, Dubai Municipality, Dubai, United Arab Emirates

**Keywords:** Type 2 Diabetes mellitus, Chronic diseases, Medication adherence, Coronavirus

## Abstract

**Background:** Patients with chronic diseases often experience difficulty adhering to recommended treatments as instructed by their healthcare professionals. Recently, diabetes has been associated with the severity of the novel coronavirus disease (Covid-19), which raises the importance of improving medication adherence for diabetic patients to enhance the right use of antidiabetics amid the Covid-19 pandemic.

**Methods:** This work assesses medication adherence among type 2 diabetes mellitus patients in the United Arab Emirates (UAE) and identifies the set of key demographic and health factors significantly associated with medication adherence. A descriptive cross-sectional study was conducted on an appropriate sample of type 2 diabetic patients in the UAE, with 180 patients of both genders and various social levels. A validated version of the eight-item Morisky Medication Adherence Scale (MMAS) was used for data collection.

**Results:**  The average MMAS score was 4.88, with 95% confidence intervals (CI) 4.6 and 5.2. 61.67% (n=111), 28.89% (n=52), and 9.44% (n=17) of patients were categorized into low, medium, and high adherent groups, respectively. These findings indicate that a high level of non-compliance to antidiabetic regimens among the population in the UAE.

**Conclusions** **: **Patients demonstrated low level of compliance to antidiabetic regimens. Therefore, they must receive up-to-date knowledge about the disease and the treatment and enable easy access to their health care providers to enhance medication adherence.

## Introduction

Medication adherence is the extent to which an individual takes medication as instructed by a healthcare professional. According to the World Health Organization (WHO), medication adherence is defined as “the degree to which the patient's behavior complies to the prescribed recommendations and instructions from the health care provider”. ^1^ In this context, the concept aims to integrate the professional medical opinion and the patient's preferences and lifestyle where there is mutual cooperation between the physician and the patient to improve the outcome of the treatment and enhance the prescribed regimen's efficacy. Although adherence to medications guarantees a maximum benefit from the treatment plan, many patients do not comply with the instructions and the issue of non-adherence to medications is progressively increasing nowadays. ^2^ There are several types of non-adherence described in the literature. The first type is called ‘primary non-adherence', in which the physician prescribes the treatment plan, but the patient does not follow the instructions from the beginning and does not initiate the plan itself. The second type of non-adherence is ‘non-persistence non-adherence', where the patients start the treatment regimen but do not complete it and stop the regimen without consulting their physicians. Several factors contribute to the development of this type. For example, lack of frequent communication between the patient and the physician, the patient feeling initial improvement after taking the primary doses of the prescribed drugs, the patient cannot afford the price of the completely prescribed regimen, or difficulty in the accessibility of the drug, especially in rare diseases that requires certain drugs. The third type of non-adherence is ‘non-confirming non-adherence' in which the patient does not strictly stick to the prescribed regimen. Instead, the patient changes the plan by altering doses or time, or even skipping some of them. ^3^

Assessing the patient's adherence is quite a challenging issue. However, there are several approaches adopted to fulfill this target. The first approach is the subjective method in which the patient, a family member, or caregiver is asked about the patient's commitment and the number of the doses taken with a specified time interval. The second method is the objective measurement, and it is achieved by counting the doses using electronic records for the medications or checking the pharmacy refill. A third method is the biochemical approach in which a non-harmful marker is added to the medication and then assessed in the organs where the drug is present or excreted, like assessing the serum or the drug's urine levels. The patient is considered compliant if compliance exceeds 80% of the prescribed plan of treatment. ^4^

Recently, diabetes has been associated with the severity of the novel coronavirus disease (COVID-19), where diabetic patients with COVID-19 are at higher risk to be admitted to intensive care unit and with higher mortality compared to non-diabetic COVID-19 patients ^5– 7^ raises the importance of improving medication adherence for diabetic patients to enhance the right use of antidiabetics amid COVID-19 pandemic. ^8^ As far as the authors of this work are aware, no published studies in the literature have studied medication adherence among diabetic patients during the COVID-19 pandemic in the United Arab Emirates (UAE). This work assesses medication adherence among type 2 diabetes mellitus patients in the UAE and identifies the set of key demographic and health factors significantly associated with medication adherence during the year of 2020.

## Methods

### Ethical statement

Institutional ethical approvals were obtained from Ajman University by the Research Ethics committee (Approval number P-F-H-2019-Nov-28) and the Saudi German Hospital in Ajman.

Signed/verbal consent was obtained from the patients before data collection. Informed consent was given to all patients who participated in the study before answering the questionnaire and their personal information was kept in a closed closet for a certain period with full privacy was obtained from all subjects involved in the study.

Verbal consent was for patients who were not willing to complete the questionnaire in the presence of the interviewer and was completed by five senior Pharmacy graduate students as trained interviewers/data collectors. Please, note that the presence of the interviewer can cause bias, and this should be avoided.

### Study design

This work is a descriptive cross-sectional study conducted among a convenience sample of type 2 diabetic patients in the UAE. A validated version of the eight-item Morisky Medication Adherence Scale (MMAS-8) was used for data collection. ^9^

### Participants

The criteria used to collect data for the research were composed of inclusion/exclusion criteria. For inclusion, the data collected was from randomly selected diabetic patients, who must be above the age of 18 years old and must understand Arabic or English. Questionnaires were available for participants at the reception of the hospital and clinics in envelopes for patients waiting to see their doctors and they were invited to take part in the study while waiting. Exclusion criteria included pregnant women, people with mental illness, and/or those who refused to participate.

In a systematic review published in 2015, an average of 65.8 percent (ranging from 38.5 to 93.1%) were found to be adherent to their diabetic medication. As such, we need to find if in our study there is a significant difference from 65.8%.

The sample size of 384 was determined by the use of Survey Monkey sample size calculator. The proportion of the targeted characteristics in the Northern UAE could not be estimated. Therefore, we used a calculation formula to yield the maximum number of required sample size. The following formula was used based on the sample required to estimate a proportion with an approximate 95% confidence level

$$n=z^{2}pq/d^{2}$$

where

n: the desired sample size

z: the standard normal deviation, set at 1.96 (corresponding to the 95% confidence level)

p: the proportion of the targeted characteristics in the region of Northern United Arab Emirates. Since there was no estimate available, it was set at 50% (or 0.50)

q: 1-p

d: absolute precision or accuracy, set at 0.05

Therefore, the research sample size is

$$n={\left(1.96\right)}^{2}\left(0.5\right)/0.05^{2}=384.16$$

n=384

Due to the current COVID-19 pandemic situation and lockdown, it was challenging to reach the targeted sample of 384. A total number of 180 participants was achieved.

### Data collection

The data was collected manually in the Saudi German Hospital in Ajman, six different clinics in Ajman and Sharjah, and via an online survey for other Northern Emirates. Physical questionnaires were available at the receptions of the hospital and clinics for participants who agreed to take part. Data collection took place from March 2020 to August 2020.

The self-reported eight-item MMAS was used to evaluate medication adherence among type 2 diabetes mellitus patients. ^26^ Questions 1 through 7 have categorical responses (yes/no). Item 8 has five-points Likert scale. All the questions except question 5 are reverse coded to avoid participants responding in the same manner to a series of questions irrespective of their content; each “No” is coded “1” and each “yes” is coded “0”. For question 8, if the participant selects “0”, the given score is “1”, while if they select response “4”, the given score is “0”. Categorical responses “1, 2, 3” are respectively coded “0.25, 0.75, 0.75”. The total MMAS-8 scores range from 0-8. The adherence level is considered low if the MMAS-8 score is less than 6 (score < 6), medium if in the range of 6-7 and high if equal to 8.

Missing total scores on multi-item instruments are equivalent to missing scores on a single-item instrument. Commonly used methods to deal with such missingness are complete-case analysis, mean imputation, or single-regression imputation. More advanced techniques that account for missing data uncertainty are multiple imputation or maximum likelihood estimation. Specific methods have also been developed for missing item scores in multi-item instruments, for example, person mean imputation, two-way imputation, response-function imputation, and multivariate normal imputation.

### Statistical analysis

The data were analyzed by International Business Machines Corporation-Statistical Package for the Social Science (IBM-SPSS) version 25 (Chicago, USA). Before statistical analysis, all the dependent and independent variables were checked for any data entry errors or missing data. The number of responses and percentages were used to describe categorical variables and means ± relative standard deviation (RSD) was used to describe continuous variables. For instance, to identify outliers the distance between data point and the center of all data points to ensure data points do not fall within three SD of the mean. The normality of variables was tested using the Kolmogorov-Smirnov and Shapiro-Wilk test. Non-normally distributed data were stated using medians, interquartile ranges (IQR) and mean ranks. Qualitative variables were summarized using frequencies and percentages. The Chi-square and Fischer Exact tests were used to compare differences in proportions of qualitative variables. Univariate and multivariate logistic regression analysis was used to determine the significant factors associated with medication adherence. The stepwise method was used for variable selection and model building. A *p*-value < 0.05 was chosen to make statistical significance decisions.

### Bias

Four sources of error have been described that can threaten the precision and accuracy (i.e., reliability) of survey results and must be evaluated by readers. The first type of error, coverage error (sampling bias), occurs when there is a discrepancy between the target population and the population from which the sample was derived. This type of error can compromise the ability to generalize study results.

Sampling error (or random error) occurs when the researcher surveys only a subset (sample) of all possible subjects within the population of interest. Sample error is reported usually as the mean ±1 standard error from the mean (SEM).

Measurement error (response bias) occurs when the collection of data is influenced by the interviewer or when the survey item itself is unclear from the respondent's point of view. Parallel forms (usually consisting of alternatively worded items placed throughout the survey) of either specific survey items or the entire survey instrument have been used to increase reliability of mail survey research.

Accurate assessment of measurement error relies on the provision of the questionnaire or tool used to collect data so that readers may analyze wording. Unfortunately, however, many articles relating to the results of survey research do not include the actual questionnaire used in the survey due to space and ownership issues.

Finally, nonresponse error (nonresponse bias) occurs when a significant number of subjects in a sample do not respond to the survey when responders differ from non-responders in a way that influences, or could influence, the results. Since the lower response rate may increase the potential for higher nonresponse bias, and in order to minimize the possibility of nonresponse error we could manage to increase the number of participants by including more patients from different clinics in more geographical areas in UAE. To minimize bias from the interviewers, five pharmacy graduate students were recruited and trained to conduct the interviews.

## Results

### Demographic characteristics

The demographic data of patients are as shown in Table 1. A total of 180 patients participated in the study due to restrictions with COVID-19. Among the 180 participants, 47.2% (n = 85) were female and 52.8% (n = 95) were male. Of the total participants, 38 (21.1%) were aged 20-29 years, 36 (20.0%) were aged 30-39 years, 30 (16.7%) were aged 40-49 years, 39 (21.7%) were aged 50-59 years, and 37 (20.6%) were aged ≥ 60 years. The study participants were predominantly married (n = 147, 81.7%). Nearly half of the participants (n = 81, 45%) hold bachelor certificates. The emirates of residence reported were: 23 (12.8%) from Dubai, 78 (43.3%) from Sharjah, 60 (33.3%) from Ajman and 19 (10.6%) from other Emirates. The majority of the participants were non-Emirati (n = 155, 86.1%). Among the participants 114 (63.3%) had health insurance coverage and 73 (40.6%) had an income in the range of < 5,000 Arab Emirates Dirhams (AED), 54 (30%) earned between 5,000-14,000 AED, 34 (18.9%) had an income between 15,000-24,000 AED and 19 (10.6%) had an income higher than 25,000 AED.

**Table 1.** Number and percentage of the questions on demographic information (n = 180).

Demographic	Groups	Frequency	Percentage
**Gender**	Female	85	47.2%
	Male	95	52.8%
**Age group**	20-29 years	38	21.1%
	30-39 years	36	20%
	40-49 years	30	16.7%
	50-59 years	39	21.7%
	≥60 years	37	20.6%
**Marital status**	Single	33	18.3%
	Married	147	81.7%
**Educational level**	Less than high school	34	18.9%
	High School Graduate	23	12.8%
	Diploma	24	13.3%
	University Bachelor or equivalent	81	45%
	Postgraduate	18	10%
**Emirate**	Dubai	23	12.8%
	Sharjah	78	43.3%
	Ajman	60	33.3%
	other Emirates	19	10.6%
**Nationality**	Emirati	25	13.9%
	Non-Emirati	155	86.1%
**Monthly income**	<5,000 AED	73	40.6%
	5,000-14,000 AED	54	30%
	15,000-24,000 AED	34	18.9%
	≥25,000 AED	19	10.6%
**Health insurance coverage**	Yes	66	36.7%
	No	114	63.3%

The results of statistical modeling showed that patient's age, marital status, type of antidiabetic medications, and being confident about taking diabetes medication were strong determinants of medication adherence among patients with type 2 diabetes mellitus. Significantly decreased medication adherence was observed in those with single marital status (OR 0.366; 95%, CI 0.139–0.962), patients aged 50-59 years (OR 0.238; 95%, CI 0.058–0.983), patients aged 40-49 years (OR 0.195; 95%, CI 0.044–0.857) and patients aged 30-39 years (OR 0.195; 95%, CI 0.035–0.648). On the other hand, significantly increased medication adherence was observed in the patients who had non-insulin antidiabetic medication therapy (OR 3.085; 95%, CI 1.125–8.457) and those who were confident about taking diabetes medication (OR 8.200; 95%, CI 1.036–64.908).

### Lifestyle and health characteristics

Table 2 shows the lifestyle and health information of the participants. Most of the participants exercise two times/week or less (n = 145, 80.6%) and 115 (63.9%) were nonsmokers.

**Table 2.** Number and percentage of the questions on health information (n=180)

Health factors	Groups	Frequency	Percentage
Exercise	Less than or equal to two times/week	145	80.6%
	More than or equal to three times/week	35	19.4%
Smoking status	Current	48	26.7%
	Former	17	9.4%
	Never	115	63.9%
Number of chronic diseases	One	112	62.2%
	Two	35	19.4%
	Three or more	33	18.3%
Years since diagnosed as diabetic	Less than 1 year	44	24.4%
	2-5 years	55	30.6%
	More than 5 years	81	45%
Family history with diabetes	Yes	122	67.8%
	No	58	32.2%
Number of anti-diabetic medications	One	90	50%
	Two	52	28.9%
	Three or more	38	21.1%
Type of anti-diabetic medications	Insulin	38	21.1%
	Non-insulin	114	63.3%
	Both	28	15.6%
Frequency of taking anti-diabetic medications	One	68	37.8%
	Two	83	46.1%
	Three or more	29	16.1%
Do you think that it is possible to develop complications if you do not take diabetes medication as instructed	Yes	120	66.7%
	No	38	21.1%
	Don’t know	22	12.2%
Are you confident about taking diabetes medication	Yes	164	91.1%
	No	16	8.9%

Of the total participants, 112 (62.2%) had one chronic disease, 35 (19.4%) had two chronic diseases, and 33 (18.3%) had ≥ three chronic diseases. The years since diagnosis as diabetic were as follows: 44 (24.4%) diagnosed as diabetic under one year ago, 55 (30.6%) within two to five years, and 81 (45%) diagnosed more than five years ago. Among the participants, 122 (67.8%) had a diabetic family history. Half of the study participants (n = 90, 50.0%) had one antidiabetic medication. The frequency of taking antidiabetic medications was as follows: 68 (37.8%) took the medication once, 83 (46.1%) twice, and 29 (16.1%) ≥ 3 times. The type of antidiabetic medication was detailed as follows: 38 (21.1%) had insulin therapy, 114 (63.3%) had non-insulin therapy, and 28 (15.6%) had both insulin and non-insulin therapy. The majority of the participants (n = 164, 91.1%) were confident about taking diabetes medications, and 120 (66.7%) believed that it is possible to develop complications if they do not take diabetes medication as instructed.

### Assessment of medication adherence

The average MMAS score was 4.88, with 95% confidence intervals of 4.6 and 5.2. Using the grading system mentioned in the Methods section, it can be claimed that the overall level of medication adherence was poor. Of the total 180 diabetic patients, (n = 111, 61.7%), (n = 52, 28.9%), and (n = 17, 9.4%) were low, medium, and high adherent groups, respectively. Table 3 presents the results of each item related to the MMAS-8.

**Table 3.** Number and percentage of the questions on Morisky Medication Adherence Scale (MMAS).

MMAS items	Yes		No	
	F	%	F	%
Do you sometimes forget to take your hyperglycemic medication	127	70.6	53	29.4
Over the past two weeks, were there any days when you did not take your hyperglycemic medicine	73	40.6	107	59.4
Have you ever cut back or stopped taking your medication without telling your doctor because you felt worse when you took it	59	32.8	121	67.2
When you travel or leave home, do you sometimes forget to bring along your diabetic medications	68	37.8	112	62.2
Did you take your hyperglycemic medicine yesterday	140	77.8	40	22.2
When you feel like your blood glucose is under control, do you sometimes stop taking your medicine	50	27.8	130	72.2
Taking medication every day is an inconvenience for some people. Do you ever feel hassled about sticking to your hyperglycemic treatment plan	79	43.9	101	56.1
How often do you have difficulty remembering to take all your hyperglycemic medication				
Never 68 (37.8%) Rarely 56 (31.1%) Sometimes 41 (22.8%) Often 4 (2.2%) Always 11 (6.1%)				

*Abbreviations:* F, frequency; %, percentages.

Table 4 shows the distribution of medication adherent groups according to demographic information. There was a statistically significant association between participants’ age and medication adherence, with low medication adherence scores among patients aged below 40 years compared to older patients (P = 0.020). Married patients were more likely to have better medication adherence scores than single participants (P = 0.018). Emirati patients were more likely to have better medication adherence scores than non-Emirati patients (P = 0.025).

**Table 4.** Medication adherence according to demographic factors.

Demographic	Groups	Medication adherence				p-value
		Total	Low	Medium	High	
**Gender**	Male	95	55(57.9%)	32(33.7%)	8(8.4%)	0.318
	Female	85	65(65.9%)	20(23.5%)	9(10.6%)	
**Age group**	20-29 years	38	24(63.2%)	14(36.8%)	0	0.020
	30-39 years	36	27(75%)	5(13.9%)	4(11.1%)	
	40-49 years	30	21(70%)	6(20%)	3(10%)	
	50-59 years	39	22(56.4%)	10(25.6%)	7(17.9%)	
	≥60 years	37	17(45.9%)	17(45.9%)	3(8.1%)	
**Marital status**	Single	33	27(81.8%)	6(18.2%)	0	0.018
	Married	147	84(57.1%)	46(31.3%)	17(11.6%)	
**Educational level**	Less than high school	34	22(64.7%)	9(26.5%)	3(8.8%)	0.642
	High School Graduate	23	15(65.2%)	5(21.7%)	3(13%)	
	Diploma	24	13(54.2%)	9(37.5%)	2(8.3%)	
	Bachelor degree	81	48(59.3%)	27(33.3%)	6(7.4%)	
	Postgraduate	18	13(72.2%)	2(11.1%)	3(16.7%)	
**Emirate**	Dubai	23	15(65.2%)	7(30.4%)	1(4.3%)	0.258
	Sharjah	78	48(61.5%)	20(25.6%)	10(12.8%)	
	Ajman	60	38(63.3%)	20(33.3%)	2(3.3%)	
	Other Emirates	19	10(52.6%)	5(26.3%)	4(21.1%)	
**Nationality**	Emirati	25	12(48%)	7(28%)	6(24%)	0.025
	Non-Emirati	155	99(63.9%)	45(29%)	11(7.1%)	
**Monthly income**	<5,000	73	44(60.3%)	23(31.5%)	6(8.2%)	0.199
	5,000-14,000	54	39(72.2%)	11(20.4%)	4(7.4%)	
	15,000-24,000	34	17(50%)	14(41.2%)	3(8.8%)	
	≥25,000	19	11(57.9%)	4(21.1%)	4(21.1%)	
**Health insurance coverage**	Yes	66	39(59.1%)	19(28.8%)	8(12.1%)	0.637
	No	114	72(63.2%)	33(28.9%)	9(7.9%)	

Table 5 shows the distribution of medication adherent groups according to lifestyle and health information. Patients who were confident about taking diabetes medication were more likely to have better medication adherence scores than those who weren’t confident about taking diabetes medication patients (P = 0.021).

**Table 5.** Medication adherence according to life style and health characteristics.

Health factors	Groups					p-value
		Total	Low	Medium	High	
**Exercise**	≤2 times/week	145	90(62.1%)	42(29%)	13(9%)	0.904
	≥3 times/week	35	21(60%)	10(28.6%)	4(11.4%)	
**Smoking status**	Current	48	30(62.5%)	15(31.3%)	3(6.3%)	0.836
	Former	17	9(52.9%)	6(35.3%)	2(11.8%)	
	Never	115	72(62.6%)	31(27%)	12(10.4%)	
**Number of chronic diseases**	One	112	73(65.2%)	31(27.7%)	8(7.1%)	0.432
	Two	35	18(51.4%)	11(31.4%)	6(17.1%)	
	Three or more	33	20(60.6%)	10(30.3%)	3(9.1%)	
**Years since diagnosed as diabetic**	Less than one year	44	29(65.9%)	12(27.3%)	3(6.8%)	0.877
	2-5 years	55	32(58.2%)	18(32.7%)	5(9.1%)	
	>5 years	81	50(61.7%)	22(27.2%)	9(11.1%)	
**Family history with diabetes**	Yes	122	74(60.7%)	34(27.9%)	14(11.5%)	0.396
	No	58	37(63.8%)	18(31%)	3(5.2%)	
**Number of anti-diabetic medications**	One	90	59(65.6%)	24(26.7%)	7(7.8%)	0.247
	Two	52	28(53.8%)	20(38.5%)	4(7.7%)	
	Three or more	38	24(63.2%)	8(21.1%)	6(15.8%)	
**Type of anti-diabetic medications**	Insulin	38	24(63.2%)	11(28.9%)	3(7.9%)	0.503
	Non-insulin	114	66(57.9%)	35(30.7%)	13(11.4%)	
	Both	28	21(75%)	6(21.4%)	1(3.6%)	
**Frequency of taking anti-diabetic medications**	One	68	42(61.8%)	19(27.9%)	7(10.3%)	0.878
	Two	83	52(62.7%)	25(30.1%)	6(7.2%)	
	Three or more	29	17(58.6%)	8(27.6%)	4(13.8%)	
**Do you think that it is possible to develop complications if you do not take diabetes medication as instructed**	Yes	120	74(61.7%)	34(28.3%)	12(10%)	0.917
	No	38	25(65.8%)	10(26.3%)	3(7.9%)	
	Don’t know	22	12(54.5%)	8(36.4%)	2(9.1%)	
**Are you confident about taking diabetes medication**	Yes	164	96(58.5%)	51(31.1%)	17(10.4%)	0.021
	No	16	15(93.8%)	1(6.3%)	0	

Table 6 displays univariate and multivariate logistic regression analysis results for the factors influencing medication adherence among patients with type 2 diabetes mellitus. The odds ratio in this table show the magnitude of the association, and their corresponding *p*-values indicate whether the association is statistically significant or not by using the cut-off values of 0.05, as mentioned in the Methods section.

**Table 6.** Univariate and multivariate regression analysis for the factor associated with medication adherence among patients with type 2 diabetes mellitus with single marital status.

Factors	Medication adherence (medium and high)							
	Univariate				Multivariate			
	OR	95% CI		P-value	OR	95% CI		P-value
Male	1.588	0.641	3.934	0.318	-	-	-	-
Single marital status	0.095	0.019	0.484	**0**.005	0.366	0.139	0.962	0.042
Non-Emirati nationality	0.442	0.122	1.597	0.213	-	-	-	-
Health insurance (yes)	1.229	0.507	2.977	**0**.648	-	-	-	-
**Age (Ref.** **20-29 years)**								
Above 60 years	0.299	0.051	1.747	0.180	-	-	-	-
50-59 years	0.172	0.031	0.967	0.046	0.238	0.058	0.983	0.047
40-49 years	0.145	0.026	0.815	0.028	0.195	0.044	0.857	0.030
30-39 years	0.148	0.028	0.780	0.024	0.150	0.035	0.648	0.011
**Education (Ref. Diploma)**								
Bachelor degree	0.519	0.149	1.816	0.305	-	-	-	-
Postgraduate	0.260	0.045	1.509	0.133	-	-	-	-
Less than high school	0.364	0.093	1.427	0.147	-	-	-	-
High school graduate	0.507	0.114	2.263	0.374	-	-	-	-
**Emirate of residence (Ref. other Emirates)**								
Dubai	0.628	0.137	2.882	0.550	-	-	-	-
Sharjah	0.651	0.194	2.187	0.487	-	-	-	-
Ajman	0.715	0.191	2.680	0.618	-	-	-	-
**Monthly income (Ref. < 5,000)**								
5,000-14,000	0.465	0.170	1.272	0.136	-	-	-	-
15,000-24,000	0.915	0.265	3.157	0.888	-	-	-	-
≥ 25,000	0.981	0.242	3.979	0.979	-	-	-	-
**Exercise (Ref. ≤ 2 times/week)**								
≥ three times/week	0.763	0.253	2.297	**0**.631	-	-	-	-
**Smoking status (Ref. current smoking)**								
Never	1.194	0.427	3.338	0.736	-	-	-	-
Former	1.507	0.336	6.765	0.593	-	-	-	-
**Number of chronic diseases (Ref.** **3** **or more)**								
Two	1.491	0.468	4.748	0.500	-	-	-	-
One	0.958	0.334	2.746	0.936	-	-	-	-
**Years since diagnosed as diabetic (Ref.** **2** **-** **5** **years)**								
More than five years	0.724	0.262	2.006	0.535	-	-	-	-
Less than one year	1.199	0.379	3.798	0.757	-	-	-	-
**Diabetic family history**	1.463	0.641	3.341	0.366	-	-	-	-
**Number of anti-diabetic medications (Ref.** **3** **or more)**								
Two	1.589	0.483	5.228	0.446	-	-	-	-
One	0.791	0.202	3.103	0.737	-	-	-	-
**Type of anti-diabetic medications (Ref. both)**								
Non-insulin	4.514	1.234	16.505	0.023	3.085	1.125	8.457	0.029
Insulin	2.491	0.600	10.351	0.209	-	-	-	-
**Frequency of taking anti-diabetic medications (Ref.** **3** **or more)**								
Two	0.607	0.193	1.912	0.394	-	-	-	-
One	0.610	0.174	2.145	0.441	-	-	-	-
**Belief of “not taking diabetes medications as instructed could results in a complications” (Ref. don’t know)**								
Yes	0.561	0.162	1.942	0.362	-	-	-	-
No	0.311	0.070	1.389	0.126	-	-	-	-
**Confident about taking diabetes medication (Ref. No)**								
Yes	8.431	0.806	88.190	**0**.042	8.200	1.036	64.908	0.046

*Notes:* P-values less than 0.05 were considered statistically significant, “-“ not included in the multivariate logistic regression model.

*Abbreviations*: OR, odds ratio; CI, confidence interval.

To select the set of factors that jointly influence the medication adherence, we used the stepwise procedure applied to the multivariate logistic regression model. The results of this procedure showed that patient's age, marital status, type of antidiabetic medications, and being confident about taking diabetes medication were strong determents of medication adherence among patients with type 2 diabetes mellitus.

Significantly decreased medication adherence was observed in those with single marital status (OR 0.366; 95%, CI 0.139–0.962), patients aged 50-59 years (OR 0.238; 95%, CI 0.058–0.983), patients aged 40-49 years (OR 0.195; 95%, CI 0.044–0.857), and patients aged 30-39 years (OR 0.195; 95%, CI 0.035–0.648).

On the other hand, significantly increased medication adherence was observed in the patients who had non-insulin antidiabetic medication therapy (OR 3.085; 95%, CI 1.125–8.457), and those who were confident about taking diabetes medication (OR 8.200; 95%, CI 1.036–64.908).

## Discussion

According to the MMAS, our study showed that the prevalence of low adherence to antidiabetic medication was 61.67%, medium adherence was 28.89%, and high adherence was 9.44%. These findings reveal a high level of non-compliance to antidiabetic regimens among the UAE people. Our results were consistent with the findings reported by Al Haj *et al*., who interviewed 446 UAE patients from February 2015 to November 2015, and concluded that 64.6% of the UAE population were non-adherent to antidiabetic medications. ^10^ They were also similar to the findings reported by Al Mazroui *et al*. about the non-compliance of UAE individuals to antidiabetic regimens, which reached almost 50% of the enrolled participants. ^11^ Furthermore, it is evident that the percentage of non-adherence to antidiabetic medication in the UAE is quite similar to the other neighboring Gulf Cooperation Council (GCC) countries. For example, several studies reported a similar level of non-adherence among Saudi population, ranging from 50% to 70% of the enrolled participants. ^10, 12– 14^ The similar results between the two countries are unsurprising, as they share many similar behavioral, cultural, and individual factors that may have contributed to the development of such a level of non-compliance. While the literature about adherence to antidiabetic medications among Arab individuals is quite minimal, several studies have discussed the same point worldwide. Kirkman *et al.*, identified the level of non-adherence among the American population as 31%. ^15^ Kalyango *et al.*, reported similar levels among Ugandan individuals (28.9%). ^12^ Other studies reported that the level of non-adherence in Malaysia ranges from 40% to 50%. ^16, 17^ In India, there were contradicting results that ranged from 30% to 60% of non-compliance. ^18, 19^ It can be seen from these results that the level of non-adherence is higher amongst the Arab population compared to Asian or American populations. One possible reason for the increased level of medication non-compliance among Arabs is that the level of diabetes, hypertension, and other chronic diseases is higher among Arab citizens, which increases the number of individuals diagnosed with these diseases and increases the probability of non-adherent individuals. Furthermore, the Arab people share other factors that directly contribute to higher levels of non-compliance among them. This may require more investigations to determine the contributing factors such as socioeconomic and lifestyle factors.

Studies have shown that several factors are associated with the increased level of non-compliance to antidiabetic medications. Our study showed that age is a significant determinant of the level of adherence. We report that the level of adherence significantly decreased with increased age up to the age of 60 years old. There was no statistically significant decrease in the level of adherence among patients older than 60 years old. These results were slightly different to Al Haj and their coworkers’ findings, who concluded that the level of adherence is increased with age. ^20^ Aloudah *et al.,* also reported a lower level of adherence among the younger population compared to the older population. ^13^ However, Mukherjee *et al*., reported similar results to our findings and concluded that the level of compliance significantly decreases with increased age. ^18^ In addition, Ahmad *et al.*, reported similar results about the impact of age on the level of adherence. ^17^ A possible explanation for the decreased level of adherence with increased age of patients is that patients can forget about their medications and several studies have reported that forgetfulness is one of the leading causes behind non-adherence. ^18, 21^ Another factor that influences the level of non-compliance is marital status. Our study showed low adherence to antidiabetic medication among unmarried patients compared to married ones. These findings were consistent with *Ahmed et al.*, ^11^ who reported similar findings of the significant effect of marital status on adherence level. Gelaw *et al.,* ^22^ and Thakrar *et al.*, concluded that married persons are more adherent to medications than single ones. ^19^ The possible reason behind this relationship is that married individuals are more supported by their families to adhere to their regimens. In addition, forgetfulness is less likely when the family reminds the patient of his/her schedule and medication time. However, the effect of marital status was not proven in all the literature that studied the topic. Several studies reported no significant relationship between marital status and drug adherence. ^12, 14, 18, 23^ These contradicting results signify that further investigations need to be conducted in order to find out the real relationship between the variables.

The regimen of antidiabetic medications was thoroughly analyzed in several publications. Studies discussed the types and number of prescribed antidiabetic agents and their effect on the level of adherence. In our study, we found out that the level of adherence is increased with non-insulin antidiabetic medications compared to the insulin group. Our results were consistent with most of the previous literature. Mukhurjee *et al.*, reported that a low adherence level is found among the patient group who were prescribed insulin only or insulin combined with oral hypoglycemic agents (OHAs) compared to people who use OHAs only. ^18^ The same findings were reported by Khan *et al.*, ^14^ Chua *et al.,* ^16^ Ahmad *et al.,* ^17^ Al Haj *et al*., ^20^ Aminde *et al.*, ^24^ and Bali *et al*. ^25^ Each of the previously mentioned studies confirmed that insulin only, or in combination with OHAs, is a significant factor in decreasing adherence and patients are more adherent when only OHAs are prescribed. A possible explanation for this finding is that people prefer to take their drugs via oral routes rather than injections. Hence, they are more adherent when taking oral pills. In addition, a combination of insulin and pills means that the patient must take two different drugs at different times; this increases non-adherence, as they have to stick to two (or more) drugs rather than only one. The previous hypothesis is supported by most of the previously mentioned papers, which reported a lower adherence level with increased numbers of medications. ^14, 17^ Despite several literature reports that supported our results, some authors reported different findings. Ahmed *et al.,* reported no significant difference between the number of prescribed drugs and adherence level ( *P* = 0.224). ^11^ Kalyango *et al.*, also reported no significant effect of either the number of administrated drugs or the administration route on the level of patient's adherence to the medications. ^11^ The mainstay of literature is consistent with our findings, which supports our hypothesis that the number of drugs and the administration route do affect patients' compliance with antidiabetic medications.

We also found that the patient's knowledge and persuasion to take the medication were significantly associated with higher adherence to the medication. These results were consistent with other literature which supported our findings and reported a higher level of adherence associated with increased patient knowledge of the drugs and the complications of the disease. ^17, 18^ The reason behind this finding is quite obvious. It is expected that patients’ adherence increases when they realize the importance of the drug on the prognosis of their condition, complications of the diseases, and the burden of their pathology on the family, community, and themselves in the first place.

Interestingly, we found no significant relationship between the level of adherence to antidiabetic medications and factors such as sex, educational level, monthly income, exercise, smoking, and the duration of diabetes. Each of these factors was discussed in one of the previously mentioned literature and one paper discussed a significant effect between each of these factors and the level of adherence. ^13^ In this study, low level of adherence is associated with young and male people, and that people with high education and monthly are more adherent to their medication. It was also found that the duration of diabetes when exceeding 10 years is also associated with low levels of adherence.

### Limitations

A possible limitation to our findings is the low number of participants enrolled in the study. Due to the current pandemic situation, we only managed to enroll 180 patients while most of the previous studies used the data of more than our number. Further investigations need to be conducted to reassess the effect of these factors on the level of patient's adherence to antidiabetic medications.

## Conclusions

Patient adherence to antidiabetic medications is crucial in maintaining low blood sugar levels. With the recent findings that link diabetes with the severity of COVID-19, it is essential to test the willingness of patients to improve adherence to their antidiabetic regimens during the pandemic. Our study showed that the prevalence of low adherence to antidiabetic medication is 61.67%, medium adherence is 28.89%, and high adherence is 9.44%. These results revealed a high degree of non-compliance to antidiabetic regimens among UAE individuals. Non-adherence can occur as a result of the patient intentionally disregarding their treatment schedule, or as a result of carelessness and/or forgetfulness, whereby patients often omit their medication from their daily routine or take the medication later than necessary. We should ensure that patients receive up-to-date knowledge about the disease and drugs, apply prescription treatment activities, provide written and oral information to the patient, and enable patients to contact their health care providers regularly to enhance medication adherence.

## Data availability

### Underlying data

The questionnaire responses and interviews for those not willing to complete the questionnaire themselves that were collected/conducted from/with the participants are not openly available to protect the confidentiality and privacy of the participants. All document files were eradicated after data analysis. De-identified questionnaire responses are available in English and Arabic. The data can be obtained by applying to the Ajman University Ethical Committee through rec@ajman.ac.ae. Alternatively, please contact the corresponding author Dr. Akram Ashames at a.ashames@ajman.ac.ae who can facilitate this process. The ethical committee will study data requests on a case-by-case basis to ensure integrity, objectivity, confidentiality, and professional behavior.

### Extended data

Mendeley data: ‘Questionnaire on medication adherence UAE’. http://dx.doi.org/10.17632/prvft3d63z.1. ^26^

Data are available under the terms of the Creative Commons Attribution 4.0 International license (CC-BY 4.0).
