# Supplementary material for: Medication adherence in type 2 diabetes mellitus patients during Covid-19 pandemic: a cross-sectional study from the United Arab Emirates
Source: F1000Res. 2021 Jun 25;10:435. Originally published 2021 Jun 2. [Version 2] doi: 10.12688/f1000research.51729.2 (PMC8649966; doi:10.12688/f1000research.51729.2)
Supplement: Peer review history

# Peer review: Reviewer response for version 2

**DOI:** 10.5256/f1000research.57707.r99879

**Published:** 2021-12-06

**Authors:** Mukhtar Ansari

**Affiliations:** 1 Department of Clinical Pharmacy, College of Pharmacy, University of Hail, Hail, Saudi Arabia

Overall, the topic of research is interesting and relevant, however, several studies have already been performed in gulf as well as other part of the world. The manuscript requires major revisions both in context as well as in grammar. It is advisable to copy edit the the manuscript after addressing the reviewers' comments. 

- **Abstract**: More details are required on how data was collected, who collected the data and how they were analyzed? Result section is missing with pertinent data from table 6. Conclusion should be based on the findings rather than generalization.
- **Introduction**: Can further be improved in terms of stating the problem statement and justification. 
- **Method**:
  - It is not clear whether Signed or verbal consent was taken.
  - Convenient sampling is a non-probability sampling, so the findings can not be generalized. However, more details are required about how convenient sampling was used.
  - It is confusing, to mention 'convenient sampling' and 'randomly selected diabetic patients'. \it is not clear whether English or Arabic version of the questionnaire was used?
  - This sentence "In a systematic review published in 2015, an average of 65.8 percent (ranging from 38.5 to 93.1%) were found to be adherent to their diabetic medication. As such, we need to find if in our study there is a significant difference from 65.8%" should go in introduction section.
  - How can the data collected manually and online be validated? 
  - Bias (Four sources of error...) is not required here. It would be meaningful to mention what approaches were used to avoid bias.
- **Results**:
  - Most of the information available in tables are repeated in text. It would be better to mention the significant finding only in the text. Also mention the table number in the text for better understanding. 
  - The heading of some of the tables need modification, e.g. Table No 2, 3 etc.
- **Discussion**: This section is more crucial. The authors have emphasized on comparisons without clear justifications based on other findings. 
- **Conclusions**: it should be concise, and should reflect the essence of study findings not the figures of findings.

Is the work clearly and accurately presented and does it cite the current literature?

Yes

If applicable, is the statistical analysis and its interpretation appropriate?

Partly

Are all the source data underlying the results available to ensure full reproducibility?

No

Is the study design appropriate and is the work technically sound?

Yes

Are the conclusions drawn adequately supported by the results?

Partly

Are sufficient details of methods and analysis provided to allow replication by others?

Partly

Reviewer Expertise:

Diabetes Mellitus, rational use of antibiotics, medication errors, Drug utilization. Pharmacy/ medical education

I confirm that I have read this submission and believe that I have an appropriate level of expertise to confirm that it is of an acceptable scientific standard, however I have significant reservations, as outlined above.

# Peer review: Reviewer response for version 2

**DOI:** 10.5256/f1000research.57707.r87153

**Published:** 2021-07-28

**Authors:** M.Mukhyaprana Prabhu

**Affiliations:** 1 Department of General Medicine, Kasturba Medical College, Manipal Academy of Higher Education, Manipal, Karnataka, India

Authors have reported non-compliance with Diabetic medicines. The study was carried out during COVID pandemic so the sample size was insufficient. this has been reported by authors as a limitation of the study.  

 The study has been well designed for hypothesis testing for noncompliance.

 Whether complex regimes resulted in non-compliance (OHA+ insulin) is not properly documented. Specific oral drugs or types of insulin (Long-acting or premixed or basal-bolus) that resulted in noncompliance has not been studied.

 Adverse events causing non-compliance (eg Hypoglycemic episode, edema due to glitazone have also been not studied. Whether COVID pandemic had a significant effect on noncompliance needs further evaluation as the previous literature was not during a pandemic. 

 authors can refer study from Ethiopia regarding non adherence during the COVID pandemic (Shimels *et al. *(2021 ^1^)).

Is the work clearly and accurately presented and does it cite the current literature?

Yes

If applicable, is the statistical analysis and its interpretation appropriate?

I cannot comment. A qualified statistician is required.

Are all the source data underlying the results available to ensure full reproducibility?

Yes

Is the study design appropriate and is the work technically sound?

Yes

Are the conclusions drawn adequately supported by the results?

Partly

Are sufficient details of methods and analysis provided to allow replication by others?

Yes

Reviewer Expertise:

Diabetes, hypertension, immunological disorders

I confirm that I have read this submission and believe that I have an appropriate level of expertise to confirm that it is of an acceptable scientific standard, however I have significant reservations, as outlined above.

## References

[1] : Magnitude and associated factors of poor medication adherence among diabetic and hypertensive patients visiting public health facilities in Ethiopia during the COVID-19 pandemic. *PLoS One* .2021;16(4) : 10.1371/journal.pone.0249222 e0249222 10.1371/journal.pone.0249222 33822807PMC8023457

# Response

**Published:** 2021-10-23

**Authors:** Akram Ashames

**Affiliations:** Ajman University, United Arab Emirates

Dear Editor,

 I appreciate the reviewer for his valuable comments. The comments are very important, however, the complex regimes resulted in non-compliance and the ADRs are beyond the scope of our work. This will be considered in future studies.

# Peer review: Reviewer response for version 1

**DOI:** 10.5256/f1000research.54923.r86528

**Published:** 2021-06-09

**Authors:** Khairi Mustafa Salem Fahelelbom

**Affiliations:** 1 Pharmaceutical Sciences, College of Pharmacy, Al Ain Uinversity, Al Ain, United Arab Emirates

Totally, the present article is well-established, the results are presented in very clear manner, and the subject is very interesting, but some minor revision should be considered. 

**Introduction**: 

- Kindly add up-to-date references to support your discussion. **More references **to previous related works can be added (Eg. Roncon *et al.* (2020 ^1^), Alromaihi *et al*. (2020 ^2^), Fang *et al.* (2020 ^3^))

**Methods:**

 For the **online survey design, **kindly mention: 

- which platform did you use for the data collection.  
- which tools did you use to distribute the online survey among the participants? 

**Results: **

- Results section is clearly and well-presented. 

**Discussion: **

- Please draw a concise conclusion from this study and present the limitations and future research and provide further information about study implications and future research. 

Is the work clearly and accurately presented and does it cite the current literature?

Yes

If applicable, is the statistical analysis and its interpretation appropriate?

Yes

Are all the source data underlying the results available to ensure full reproducibility?

Yes

Is the study design appropriate and is the work technically sound?

Yes

Are the conclusions drawn adequately supported by the results?

Yes

Are sufficient details of methods and analysis provided to allow replication by others?

Yes

Reviewer Expertise:

Pharmaceutical analysis

I confirm that I have read this submission and believe that I have an appropriate level of expertise to confirm that it is of an acceptable scientific standard.

## References

[1] : Diabetic patients with COVID-19 infection are at higher risk of ICU admission and poor short-term outcome. *J Clin Virol* .127: 10.1016/j.jcv.2020.104354 104354 10.1016/j.jcv.2020.104354 32305882PMC7195018

[2] : Sustainable diabetes care services during COVID-19 pandemic. *Diabetes Res Clin Pract* .2020;166: 10.1016/j.diabres.2020.108298 108298 10.1016/j.diabres.2020.108298 32623031PMC7332426

[3] : Are patients with hypertension and diabetes mellitus at increased risk for COVID-19 infection?. *The Lancet Respiratory Medicine* .2020;8(4) : 10.1016/S2213-2600(20)30116-8 10.1016/S2213-2600(20)30116-8 32171062PMC7118626
